# Systematic analysis of myocardial immune progression in septic cardiomyopathy: Immune-related mechanisms in septic cardiomyopathy

**DOI:** 10.3389/fcvm.2022.1036928

**Published:** 2023-02-24

**Authors:** Dunliang Ma, Xianyu Qin, Zhi-an Zhong, Hongtao Liao, Pengyuan Chen, Bin Zhang

**Affiliations:** ^1^Guangdong Provincial People’s Hospital’s Nanhai Hospital, The Second People’s Hospital of Nanhai District, Foshan, China; ^2^Department of Thoracic Surgery, The Sixth Affiliated Hospital, Sun Yat-sen University, Guangzhou, China; ^3^Department of Cardiology, Guangdong Cardiovascular Institute, Guangdong People’s Hospital, Guangzhou, China

**Keywords:** septic cardiomyopathy, immune infiltration, gene set enrichment analysis (GSEA), weighted correlation network analysis (WGCNA), LCN2, PTX3

## Abstract

**Background:**

The immune infiltration and molecular mechanisms underlying septic cardiomyopathy (SC) have not been completely elucidated. This study aimed to identify key genes related to SC and elucidate the potential molecular mechanisms.

**Methods:**

The weighted correlation network analysis (WGCNA), linear models for microarray analysis (LIMMA), protein-protein interaction (PPI) network, CIBERSORT, Kyoto Encyclopedia of Genes and Genomes pathway (KEGG), and gene set enrichment analysis (GSEA) were applied to assess the key pathway and hub genes involved in SC.

**Results:**

We identified 10 hub genes, namely, LRG1, LCN2, PTX3, E LANE, TCN1, CLEC4D, FPR2, MCEMP1, CEACAM8, and CD177. Furthermore, we used GSEA for all genes and online tools to explore the function of the hub genes. Finally, we took the intersection between differential expression genes (DEGs) and hub genes to identify LCN2 and PTX3 as key genes. We found that immune-related pathways played vital roles in SC. LCN2 and PTX3 were key genes in SC progression, which mainly showed an anti-inflammatory effect. The significant immune cells in cardiomyocytes of SC were neutrophils and M2 macrophages.

**Conclusion:**

These cells may have the potential to be prognostic and therapeutic targets in the clinical management of SC. Excessive anti-inflammatory function and neutrophil infiltration are probably the primary causes of SC.

## 1. Introduction

Researchers recognize sepsis as a life-threatening condition that is caused by a dysregulated host response to infection (1). It is the immune response of the organism to pathogens and immunogenic substances, causing autoimmune damage. Sepsis is common in severe health conditions, and the development of sepsis may lead to septic shock and multiple organ dysfunction syndromes (MODS), which once occurs, mortality can be up to 28–56%. The heart is the main target organ of sepsis, and more than 50% of patients with severe sepsis have myocardial dysfunction, which is called septic cardiomyopathy (SC) (2). However, due to a lack of uniform diagnostic criteria, the prevalence of SC varies in different reports. Beesley et al. (3) reported the incidence of myocardial dysfunction in sepsis patients as ranging from 10 to 70%.

Inflammatory responses and immune cell infiltration widely exist in many types of cardiomyopathy. For example, in heart tissue with diabetic cardiomyopathy, inflammatory responses have been found to be significantly activated, as manifested by infiltration of multiple immune cells, increased cytokines, and multiple chemical factors (4). Similar results have also been confirmed in animal models (5, 6). In the state of diabetes mellitus, macrophages may induce tissue infiltration, transform into the proinflammatory phenotype of M1, and be associated with the activation of inflammatory signaling pathways in leukocytes (7). Thus, inflammatory responses are closely related to cardiac function. Myocardial dysfunction can increase sepsis-induced mortality, but no reports have elucidated the underlying pathophysiological mechanisms of SC. The molecular mechanism that may be involved in the pathogenesis of SC remains to be studied, and there is a need to screen potential targets for the treatment of SC. Among the pathogenic factors contributing to SC, the imbalanced inflammatory responses caused by sepsis directly correlate with the dysfunction of myocardial cells. Previous studies have reported that sepsis begins with the host immune system’s response to invasive pathogens, eventually leading to activation of the innate immune response (8). Bacterial products, including endotoxins and exotoxins, can directly or indirectly stimulate various target cells, including monocytes, polymorphonuclear neutrophils, or endothelial cells, thereby causing inflammation (9). Endotoxins and exotoxins, through varied signal transduction pathways, activate both positive and negative feedback loops within the immune system. Sepsis-induced dysregulation of the normal immune response can lead to a variety of harmful effects, including SC. Therefore, a thorough understanding of the molecular immune mechanism involved in the pathogenesis of SC could be one of the breakthroughs that may help in the treatment of SC in the future.

In this study, we downloaded the gene expression profile (GSE79962) deposited by Matkovich et al. (10) from Gene Expression Omnibus (GEO) databases to uncover further the biomarkers associated with SC development and progression. We aimed to identify key genes related to SC, as well as to further elucidate the potential molecular mechanisms through bioinformatics analysis.

## 2. Materials and methods

### 2.1. Data

**FIGURE 1:**
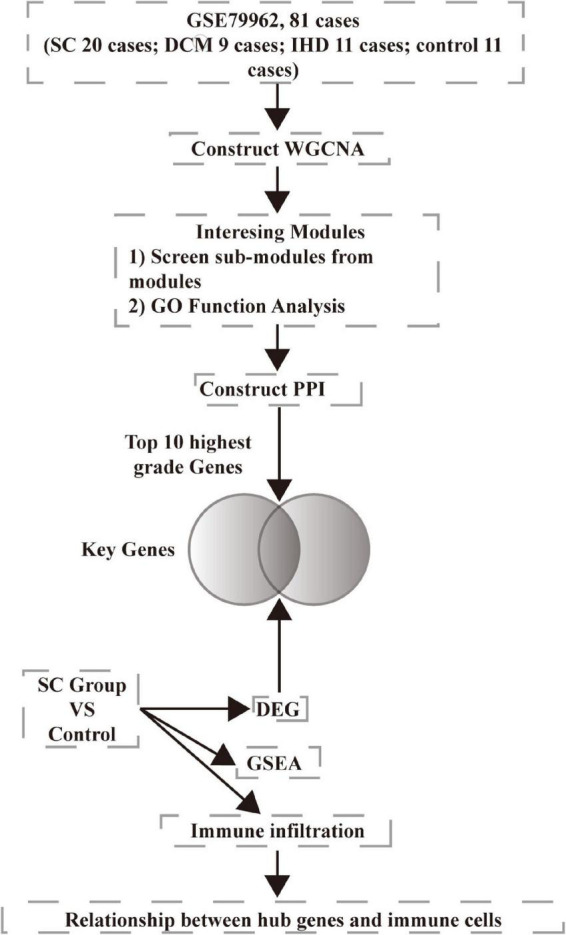
Workflow used for bioinformatics analyses.

We downloaded microarray data GSE79962 from the NCBI Gene Expression Omnibus database (GEO).^1^ The data involved ischemic heart disease (IHD, 11 samples), non-failing heart (control, 11 samples), dilated cardiomyopathy (DCM, 9 samples), and septic cardiomyopathy (SC, 20 samples). We chose all the samples in the study. We downloaded the annotation information of the microarray, GPL6244, Affymetrix Human Gene 1.0 ST Array [transcript (gene) version]. We preprocessed the raw data using R version 3.6.0. The analysis workflow is presented in Figure 1.

### 2.2. WGCNA network construction

We constructed co-expression networks using the weighted correlation network analysis (WGCNA) package in R (11). We did not filter genes. We imported gene expression values into WGCNA to create co-expression modules using the automatic network construction function blockwiseModules with default settings. We set the power value by the condition of scale independence as 0.9. The unsigned TOMType mergeCutHeight was 0.25, and the minModuleSize was 50.

### 2.3. Module and gene selection

To find biologically or clinically significant modules for SC, module eigengenes were used to calculate the correlation coefficient with samples. We calculated the intramodular connectivity (function softConnectivity) of each gene. We thought genes with high connectivity tended to be hub genes which might have essential functions. We imported the positive correlation modules into Cytoscape software (version 3.7.1), using the MCODE plugin, setting the degree cutoff at no less than 10, to screen the key sub-modules.

### 2.4. Functional analysis of module genes

Because the three modules were all positively correlated to SC, we imported all genes from these three sub-modules into the STRING database (version 11.0).^2^ We obtained the results of gene ontology (GO) enrichment analysis, Kyoto Encyclopedia of Genes and Genomes (KEGG) pathway enrichment analysis, and protein-protein interaction (PPI). Significantly enriched GO terms and pathways in genes in a module compared to the background were defined by hypergeometric tests and a threshold of a minimum required interaction score 0.700 (high confidence). After that, we imported a PPI network into Cytoscape software (version 3.7.1), using the cytoHubba plugin to screen the hub genes by 12 methods. We performed functional analysis and disease prediction of hub genes through the online tools Metascape database^3^ and ToppGene database.^4^ We used the “limma” package of the R language to identify the differential expression genes (DEGs) between the SC and control groups, according to the adjusted *p*-value < 0.05 and |logFC| > 0.7. We screened the shared genes between DEGs and hub genes as key genes.

### 2.5. GSEA and immune infiltration analysis using the CIBERSORT method

Meanwhile, we performed the gene set enrichment analysis (GSEA) with hallmark gene sets using the 11 control samples and 20 SC samples according to the default values. The criteria of significant results were set as normal enrichment score (|NES| > 1), normal *p*-value < 0.05, and FDR < 0.25. To characterize the immune cell subtypes in SC progression, we applied the CIBERSORT estimate software^5^ to quantify the immune cell fractions for the gene expression matrix derived from SC samples. Then, we identified the correlation between the hub genes and immune cell subtypes.

## 3. Results

### 3.1. Screening the key modules in the network

**FIGURE 2:**
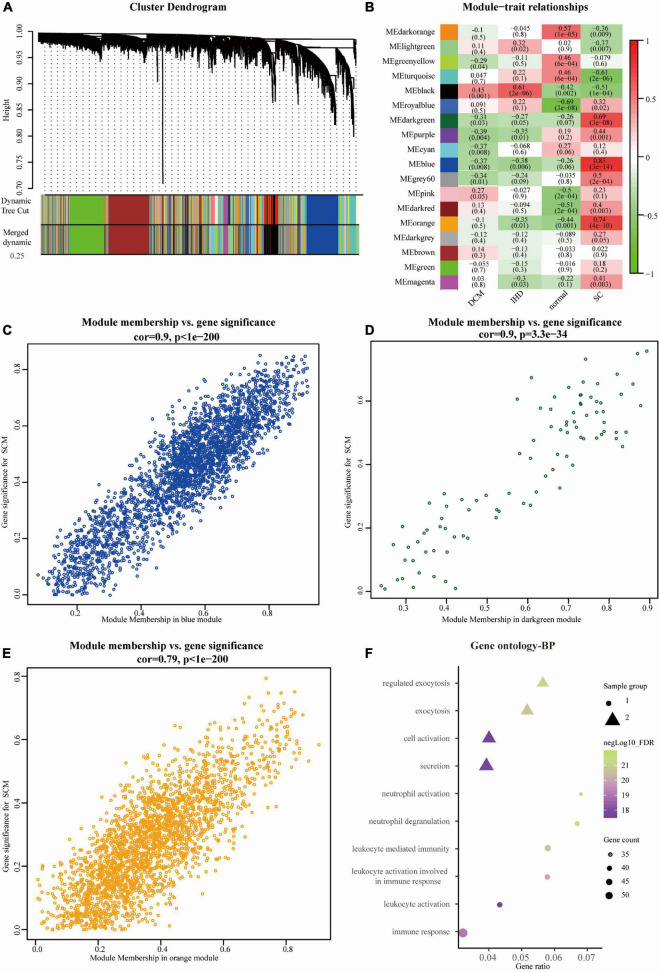
Overview of WGCNA network construction of all genes **(A)** Gene modules’ dendrogram plots of all genes; **(B)** module-trait relationships of four groups in 18 modules. **(C–E)** Module membership vs. gene significance between three significant modules, including blue module (Pearson cor = 0.9, *p* < 1e-200), dark green module (cor = 0.9, *p* = 3.3e-34), and orange module (Pearson cor = 0.79, *p* < 1e-200); **(F)** the bubble diagram showing the GO (biological process, BP) function enrichment of genes in sub-modules. The size represents the gene counts, and node colors show the gene expression negative Log10_FDR (false discovery rate).

Expression data of 18,818 genes in the 51 samples were screened. These samples included SC, control (non-failing donor heart), ischemic heart disease (IHD), and dilated cardiomyopathy (DCM). They were used to construct the co-expression modules with the WGCNA algorithm. Following the data preprocessing, we set the power value. The power value was four when the condition of scale independence was 0.9 (Supplementary Figure 1). We clustered genes into 26 correlated modules. We tried to identify sample-associated co-expression modules using WGCNA (Figures 2A, B). At last, we got 18 co-expression modules, which were illustrated in the branches of a dendrogram with different colors. We focused just on the SC group. Therefore, we chose the dark green module (Pearson cor = 0.69, *p* = 3e–8), blue module (Pearson cor = 0.83, *p* = 3e–14), and orange module (Pearson cor = 0.74, *p* = 4e–10), as moderately or more positively related with SC. The number of genes in the three modules was 2652 (blue), 73 (dark green), and 2,041 (orange). The information about the genes in the three modules is listed in Supplementary Table A1. The relationship of module membership to gene significance in the modules showed was cor = 0.9 and *p* < 1e-200 in the blue module (Figure 2C), cor = 0.9 and *p* = 3.3e-34 in the dark green module (Figure 2D), and cor = 0.79 and *p* < 1e-200 in the orange module (Figure 2E). We imported the three modules into the Cytoscape software and used the MCODE to screen the three sub-modules, setting the criteria of the MCODE score to more than 10. After screening, we got 58 genes from the orange module (average MCODE score = 33.98), 37 genes from the blue module (average MCODE score = 14.23), and 53 genes from the dark green module (average MCODE score = 41.16) (Supplementary Table A2).

### 3.2. Functional enrichment analysis of genes in critical modules

We imported all of the screened genes (a total of 148 genes) from the three modules into the STRING database to construct a PPI network. Meanwhile, we got GO enrichment analysis results according to a false discovery rate (FDR) < 0.05.

**FIGURE 3:**
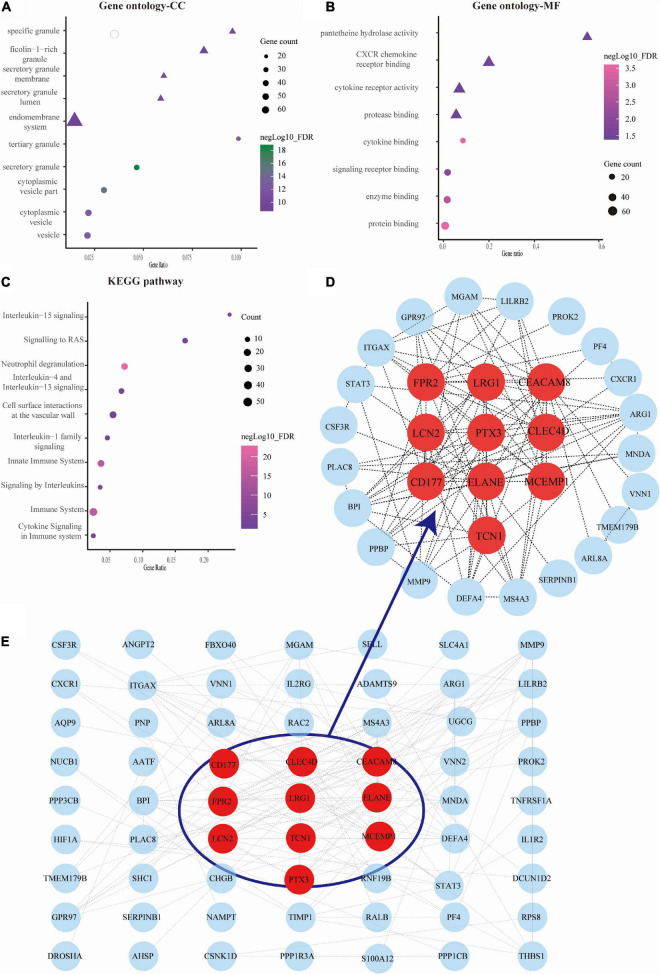
Analysis of gene ontology (GO) function, Kyoto Encyclopedia of Genes and Genomes (KEGG) pathways, and protein-protein interaction (PPI) network of genes in septic cardiomyopathy (SC)-related modules. **(A–C)** The bubble map showing the GO function (cellular component, CC, and molecular function, MF) and KEGG pathways were constructed by the STRING database. The sizes represent negative Log10 (FDR). **(D)** The gene PPI network was also constructed based on the STRING database. **(E)** The plots showing the top 10 higher degree hub genes for SC.

We obtained the top 10 biological process (BP) terms, including neutrophil activation (FDR = 1.29E–22), neutrophil degranulation (FDR = 2.65E–22), regulated exocytosis (FDR = 6.34E–22), exocytosis (FDR = 2.02E–21), leukocyte mediated immunity (FDR = 2.20E–21), leukocyte activation involved in immune response (FDR = 9.78E–21), immune response (9.07E–20), leukocyte activation (FDR = 1.86E–18), secretion (FDR = 1.86E–18), and cell activation (FDR = 2.56E–18) (Figure 2F). We obtained GO terms of the top 10 cellular components (CC), consisting of secretory granule (FDR = 2.49E–19), cytoplasmic vesicle part (FDR = 1.34E–15), cytoplasmic vesicle (FDR = 4.74E–13), vesicle (FDR = 4.74E–13), tertiary granule (FDR = 4.74E–13), specific granule (FDR = 3.63E–11), secretory granule lumen (FDR = 6.73E–11), secretory granule membrane (FDR = 1.42E–10), ficolin-1 rich granule (FDR = 1.82E–10), and endomembrane system (FDR = 1.33E–09) (Figure 3A). Meanwhile, we harvested GO terms of 8 molecular functions (MFs), comprising protein binding (FDR = 0.00028), cytokine binding (FDR = 0.00028), enzyme binding (FDR = 0.0018), signaling receptor binding (FDR = 0.0124), CXCR chemokine receptor binding (FDR = 0.0297), protease binding (FDR = 0.0361), cytokine receptor activity (FDR = 0.0361), and pantetheine hydrolase activity (FDR = 0.0361; Figure 3B). Regarding the KEGG pathway enrichment, the genes were significantly enriched in pathways including neutrophil degranulation (FDR = 4.58E–23), innate immune system (FDR = 1.30E–16), immune system (FDR = 1.30E–16), signaling by interleukins (FDR = 8.52E–06), cytokine signaling in immune system (FDR = 0.00025), and others (Figure 3C and Supplementary Table A3).

We used Cytoscape software to visualize the PPI network from the STRING database, which is shown in Figure 3D. Through the cytoHubba plugin, we exported the results of 12 algorithms and screened the top 10 genes as hub genes. These included LRG1, LCN2, PTX3, ELANE, TCN1, FPR2, CLEC4D, MCEMP1, CEACAM8, and CD177 (Figure 3E and Supplementary Table A4).

### 3.3. GSEA and immunocyte infiltration analysis

**FIGURE 4:**
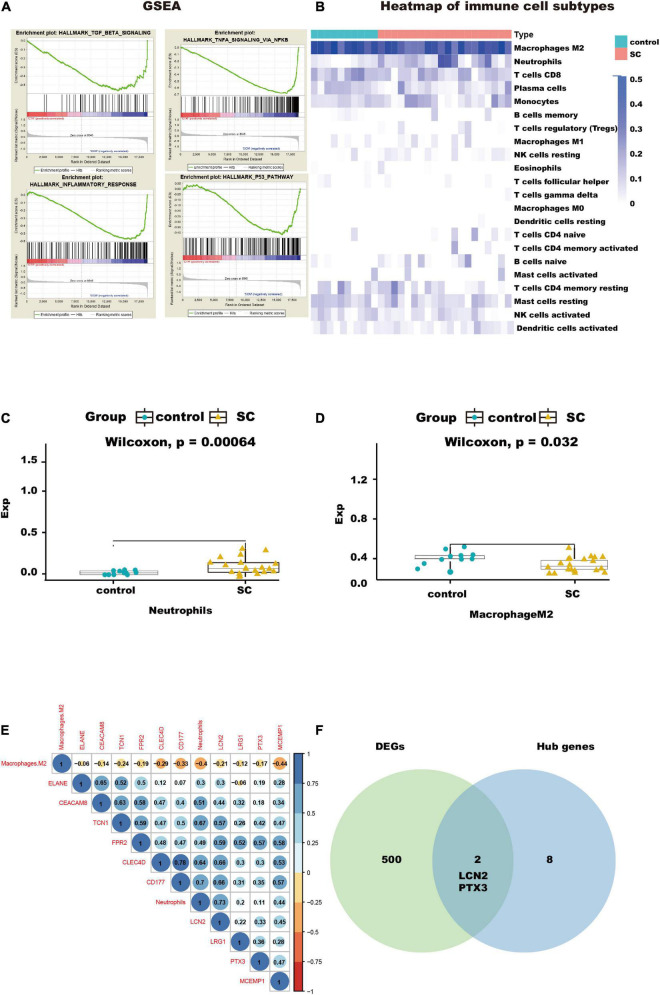
Immunocyte infiltration analysis and potential immunocyte subtype detection. **(A)** This diagram shows the immune-related pathways by gene set enrichment analysis (GSEA) analysis. **(B)** This heatmap shows the immunocyte infiltration difference between SC and control heart samples. **(C,D)** Box plots presenting significantly infiltrated immunocyte subtypes, neutrophils, and M2 macrophages. **(E)** This correlated heatmap shows the relationship between immunocytes and hub genes. **(F)** Intersection between DEGs and hub genes, identifying the key genes, LCN2, and PTX3.

The results of GO and KEGG enrichment analysis also indicated that immune and inflammatory events played a vital role in cardiac tissue in SC. Furthermore, the results of GSEA with hallmark gene sets between the control and SC indicated significant differences in the myocardium of SC, such as TGF-beta signaling, TNF-alpha signaling through NF-kappa B, inflammatory response, and TNF and P53 pathways (we set the criteria |NES| > 1, NOM *p* < 0.01, FDR < 0.25; Figure 4A and Table 1). To characterize the immunocyte status of cardiac tissues in SC, we performed a tissue immune infiltration analysis. We found that there was no significant difference in 22 immune subtypes in the myocardium of SC and the control, except for M2 macrophages (Wilcoxon, *p* = 0.032) and neutrophils (Wilcoxon, *p* = 0.00064; Figures 4C, D). A heatmap of immune cell subtypes is illustrated in Figure 4B.

**TABLE 1:** The top five pathways enriched by all genes through gene set enrichment analysis (GSEA) (|NES| > 1, Nom *p* < 0.01, and FDR < 0.25), GSEA (gene set enrichment analysis).

Name	NES	Nom *p*-value	FDR *q*-value
HALLMARK_TGF_ BETA_SIGNALING	−1.76045	0.00998	0.18903
HALLMARK_TNFA_ SIGNALING_*VIA*_ NFKB	−1.65812	0.001961	0.242987
HALLMARK_P53_ PATHWAY	−1.63801	0.005725	0.19739328
HALLMARK_IL2_ STAT5_SIGNALING	−1.5659359	0.003937008	0.1933887
HALLMARK_ ESTROGEN_ RESPONSE_LATE	−1.4913071	0.007797271	0.18019646

### 3.4. Identification of the relationship between hub genes and key immune cell subtypes in SC

We found that two subtypes of immune cells in the infiltration were significant. These were neutrophils and M2 macrophages. Importantly, the neutrophils had the highest relative infiltration value. We analyzed the relationship between the 10 hub genes in immune-related pathways and the two immune cell subtypes and found the neutrophils had positive correlations with all hub genes, especially with CLEC4D, CD177, LCN2, and TCN1, but M2 macrophages had negative correlations with all hub genes and neutrophils; Figure 4E). This suggests that to some extent, neutrophils can promote the progression of SC in the myocardium, but that M2 macrophages do the opposite.

### 3.5. Investigating the functional role of hub genes and identification of key genes

To further understand how the hub genes were correlated with SC, we applied the Metascape online database to explore their biological functions. The results of the top five GO term enrichments in hub genes included neutrophil degranulation, neutrophil activation involved in immune response, neutrophil activation, neutrophil mediated immunity, and granulocyte activation. The results showed all hub genes are involved in the neutrophil and immune response (Table 2). We explored enriched pathways of hub genes involved in neutrophil degranulation, the innate immune system, antimicrobial peptides, and similar topics (Supplementary Table A5). We also predicted diseases related to hub genes using the ToppGene database. These diseases included sepsis, immune neutropenia, septicemia, and others (Table 3 and Supplementary Table A5). We found 467 DEGs between the SC group and the control (Supplementary Table A6). Finally, we took the intersection between DEGs and hub genes to identify LCN2 and PTX3 as key genes (Figure 4F). As for the key genes, we compared the expression levels of LCN2 and PTX3 in different kinds of myocardial injury from these samples. We found LCN2 and PTX3 had significantly higher expression in the SC group (Supplementary Figure 2).

**TABLE 2:** The top five GO items enriched by hub genes, GO (gene ontology).

Category	Name	*q*-value	Enriched genes
GO: BP	Neutrophil degranulation	9.59752E–05	CD177, CEACAM8, CLEC4D, ELANE, FPR2, LCN2, LRG1, MCEMP1, PTX3, TCN1
GO: BP	Neutrophil activation involved in immune response	9.59752E–05	CD177, CEACAM8, CLEC4D, ELANE, FPR2, LCN2, LRG1, MCEMP1, PTX3, TCN1
GO: BP	Neutrophil activation	9.59752E–05	CD177, CEACAM8, CLEC4D, ELANE, FPR2, LCN2, LRG1, MCEMP1, PTX3, TCN1
GO: BP	Neutrophil mediated immunity	9.59752E–05	CD177, CEACAM8, CLEC4D, ELANE, FPR2, LCN2, LRG1, MCEMP1, PTX3, TCN1
GO: BP	Granulocyte activation	9.59752E–05	CD177, CEACAM8, CLEC4D, ELANE, FPR2, LCN2, LRG1, MCEMP1, PTX3, TCN1

**TABLE 3:** The top five diseases enriched by hub genes.

ID	Name	*p*-value	FDR	Enriched genes
C0243026	Sepsis	1.73E–07	7.56E–05	FPR2, CLEC4D, CD177, ELANE, LCN2, PTX3
C0272175	Immune neutropenia	2.04E–06	4.45E–04	CD177, ELANE
C0036690	Septicemia	3.99E–06	5.81E–04	FPR2, CD177, ELANE, LCN2, PTX3
C0085578	Thalassemia minor	3.08E–05	3.36E–03	CD177, LCN2
C0005283	Beta thalassemia	4.51E–05	3.74E–03	CD177, ELANE, LCN2

## 4. Discussion

In this study, we screened key modules in SC by analyzing a public dataset (GSE79962). Compared with the control group, IHD group, and DCM group, a total of three modules were positively correlated with SC. These included the orange module, blue module, and dark green module. We chose 148 genes from the three modules using the MCODE plugin of Cytoscape for functional enrichment analysis. Notably, we found most genes in the three modules were enriched in the immune response, leukocyte activation, neutrophil degranulation, and similar events.

Regarding the KEGG pathway enrichment, the genes were significantly enriched in immune-related pathways, including neutrophil degranulation, the innate immune system, the immune system, and cytokine signaling in the immune system. The results of GSEA with hallmark gene sets between control and SC indicated that significantly different pathways in the myocardium of SC were immune-related. Neutrophils degranulated during phagocytosis, releasing a series of lysosomal enzymes, which caused damage to blood vessels and surrounding tissues, leading to cardiac dysfunction (12). Sepsis leads to an auto-amplifying cytokine production known as the cytokine storm. At the same time, activation of Toll-like receptors (TLRs) releases a large number of inflammatory cytokines, such as TNF, IL-1, interferon regulatory factor 3 (IRF3) (13). The activation of these immune responses leads to damage of myocardial tissue and cardiac dysfunction. Previous studies have reported that TLRs can attenuate SC through activation of innate immune and inflammatory responses (14, 15). TLR is a kind of pattern recognition receptor that can activate the innate immunity response, playing a critical role through activation of NFκB which is an important transcription factor controlling the expression of inflammatory cytokine genes (16). TLRs play a major role in the pathophysiology of cardiac dysfunction during sepsis (14). In an animal model, TLR2 was found that can influence cardiac function through deteriorating sarcomere shortening (17, 18). TLRs deficiency attenuated cardiac dysfunction in a mouse model through inhibition of sepsis-induced activation of TLR4 mediated NF-κB signaling pathways, and prevention of the macrophage and neutrophil infiltration. In addition, lipopolysaccharide (LPS) has been demonstrated to induced macrophage inflammation through TLRs, leading to the release of proinflammatory cytokines (19). In patients with sepsis, increased serum lactate levels increased mortality through activation of innate immune and inflammatory responses (20, 21). Through the CIBERSORT method, we found that the infiltration value of neutrophils and M2 macrophages in the myocardium of SC is significant. Therefore, we found that immunity and inflammation play important roles in myocardial dysfunction in SC. In this condition, neutrophils were positively correlated with immune-related genes, and M2 macrophages were negatively correlated with immune-related genes.

Macrophages are the “frontier soldiers” of innate immunity. The function of macrophages is classified into two types, type M1 (classically activated) and type M2 (alternatively activated). Type M1 macrophages can secrete chemokines for a proinflammatory function, and type M2 macrophages mainly secrete chemokines in the late stage of inflammation to play an anti-inflammatory role (22, 23). M2 macrophages mainly promote tissue remodeling and repair, and previous studies showed that an increase in M2 macrophage infiltration in myocardium promotes fibrosis (23–25). The decrease of M2 macrophages in SC leads to a reduction of anti-inflammatory chemokines and supports the progression of SC. The polarization of the M1 macrophage is mainly regulated by transcription factors IRF5 and STAT1, and the M2 macrophage is regulated by IRF4 and STAT6 (26). Many immunomodulators can promote M1 macrophage polarization, such as IFN, TNF, IL-1, IL-6, LPS, B-cell activator (BAFF), and proliferation-inducing ligand (APRIL) (26–29). In addition, some metabolites, such as saturated fatty acids and oxidized lipoproteins, can also induce M1 macrophage polarization (27). Similarly, inflammatory factors, such as IL-4, IL-13, IL-10, IL-33, and TGF, as well as metabolites such as unsaturated fatty acids and retinoic acids, can induce M2 macrophage polarization (30, 31). Likewise, neutrophils are key factors in the immune response to sepsis. Under normal conditions, neutrophils control infection, but excessive stimulation or dysregulated neutrophil functions are believed to be responsible for sepsis pathogenesis (12). In SC, we found significant neutrophil infiltration in cardiac tissues.

We screened 10 hub genes from the PPI network constructed from 148 genes. These 10 genes are also involved in several immune-related pathways directly, which include LRG1, LCN2, PTX3, ELANE, TCN1, CLEC4D, FPR2, MCEMP1, CEACAM8, and CD177. Next, we used online tools (the ToppGene and Metascape databases) to explore the function of the hub genes. The results showed that the hub genes were related to immune-related pathways and diseases. Among these genes, LRG1 is highly correlated with neutrophils and other genes composed of TCN1, FPR2, CLEC4D, and CD177. LRG1 is expressed during granulocyte differentiation. From the GeneCards database,^6^ we found that the super pathway for LRG1 is the innate immune system. BP terms included response to bacterium, positive regulation of transforming growth factor-beta receptor signaling pathway, neutrophil degranulation, and similar terms. Recombinant human LRG is used as a diagnostic aid in acute appendicitis (32). Similarly, LRG1 may be used as a diagnostic marker for SC.

In our research, we found that LCN2 and PTX3 in 10 hub genes existed in DEGs, as key genes. LCN2, encoding the lipocalin-2 (also known as neutrophil gelatinase-associated lipocalin), is a critical iron regulatory protein during physiological and inflammatory conditions and exerts mostly a protective role in inflammatory bowel diseases and urinary tract infection by limiting bacterial growth (33, 34). In the heart, some reports also indicated that LCN2 was significantly expressed during *in vivo* and *in vitro* experiments on cardiac hypertrophy and heart failure, and high plasma LCN2 was correlated with high mortality and myocardial dysfunction in severe sepsis (35, 36). Nevertheless, Guo et al. (37) found that LCN2^–/–^ mice displayed an up-regulation of M1 macrophages but down-regulation of M2 macrophages. These mice had profound up-regulation of proinflammatory cytokines, suggesting that LCN2 plays a role as an anti-inflammatory regulator in macrophage activation. Overexpression of LCN2 is consistent with down-regulation of M2 macrophages in SC. PTX3 plays a role in the regulation of innate resistance to pathogens and inflammatory reactions. Paeschke et al. (38) showed that inflammatory injury of heart tissue was aggravated in mice when PTX3 was knocked down. Yamazaki et al. (39) demonstrated that bacterial LPS, induced expression of anti-microbial glycoproteins-PTX3 and LCN2 in macrophages. Therefore, we concluded that LCN2 and PTX3 might lead to excessive anti-inflammatory effects for SC progression.

Our study has some limitations. First, the sample size of this study is relatively small and additional clinical samples are necessary. However, we barely obtain more, due to the difficulty in obtaining SC samples. Besides, despite that LCN2 and PTX2 are related to neutrophil function as reported, there is no direct evidence to validate that LCN2 and PTX3 are involved in the progression of SC. Simultaneously, the underlying mechanism by which LCN2 and PTX3 affect SC remains unclear. Last but not the least, although our conclusion is based on bioinformatics analysis, more experimental results will help to increase the reliability of the results. We expect further understanding of the regulatory functions of key genes on SC through other means.

## 5. Conclusion

To sum up, we found that genes in three modules played vital roles in the immune-related pathways. LCN2 and PTX3 were key genes in SC progression and mainly showed anti-inflammatory effects. The significant immune cells in cardiomyocytes of SC were neutrophils and M2 macrophages. Therefore, LCN2 and PTX3 may have the potential to perform as prognostic and therapeutic targets in the clinical management of SC. Excessive anti-inflammatory function and neutrophil infiltration were probably the primary causes of SC, but this needs further analysis.

## Data availability statement

The datasets presented in this study can be found in online repositories. The names of the repository/repositories and accession number(s) can be found in the article/Supplementary material.

## Author contributions

DM, XQ, and Z-AZ analyzed the data and drafted the manuscript. HL and PC edited the manuscript. BZ supervised the project, gave advice regarding the project design, and edited the manuscript. All authors contributed to the article and approved the submitted version.

## Footnotes

1
http://www.ncbi.nlm.nih.gov/geo/

2
https://string-db.org/

3
http://metascape.org/

4
https://toppgene.cchmc.org/

5
https://cibersort.stanford.edu/

6
https://www.genecards.org/

## Supplementary material

Supplementary Figure 1 The chosen soft threshold power of genes matrix. **(A)** The correlation between scale free topology fit index and powers. The cutline was 0.9. **(B)** The correlation between mean connectivity and soft powers.

Supplementary Figure 2 LCN2 and PTX3 had significantly higher expression in the SC group. Expressions of LCN2 **(A)** and PTX3 **(B)** in control (*n* = 11), ICM (*n* = 11), DCM (*n* = 9), and SC group (*n* = 20).
