# Supplementary figures and images for: Systematic analysis of myocardial immune progression in septic cardiomyopathy: Immune-related mechanisms in septic cardiomyopathy
Source: Front Cardiovasc Med. 2023 Feb 24;9:1036928. doi: 10.3389/fcvm.2022.1036928 (PMC10002421; doi:10.3389/fcvm.2022.1036928)

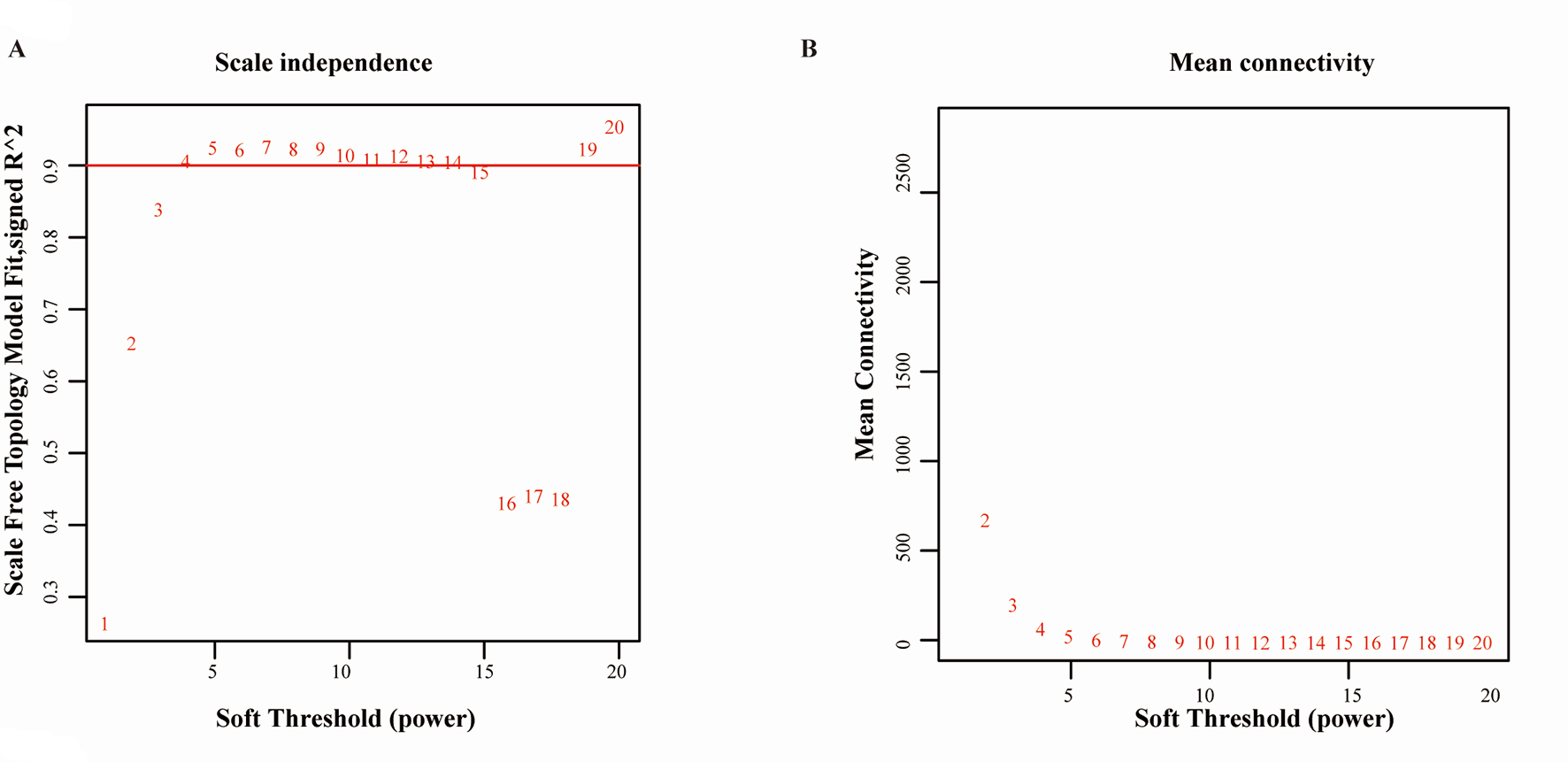

Supplement: Supplementary Figure 1 — The chosen soft threshold power of genes matrix. (A) The correlation between scale free topology fit index and powers. The cutline was 0.9. (B) The correlation between mean connectivity and soft powers. [file Image_1.tiff]

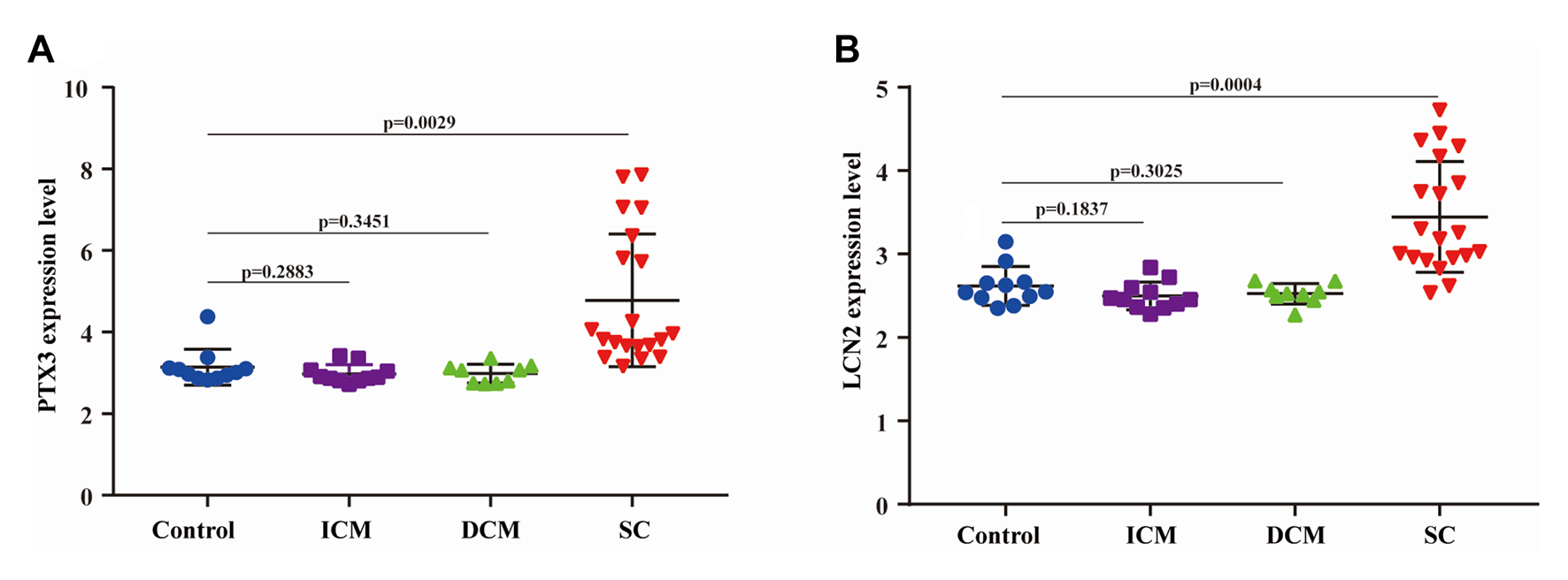

Supplement: Supplementary Figure 2 — LCN2 and PTX3 had significantly higher expression in the SC group. Expressions of LCN2 (A) and PTX3 (B) in control (n = 11), ICM (n = 11), DCM (n = 9), and SC group (n = 20). [file Image_2.tif]
